# Inertial Sensor Based Analysis of Lie-to-Stand Transfers in Younger and Older Adults

**DOI:** 10.3390/s16081277

**Published:** 2016-08-12

**Authors:** Lars Schwickert, Ronald Boos, Jochen Klenk, Alan Bourke, Clemens Becker, Wiebren Zijlstra

**Affiliations:** 1 Department of Clinical Gerontology, Robert-Bosch Hospital, Stuttgart 70376, Germany; ronald.boos@googlemail.com (R.B.); jochen.klenk@rbk.de (J.K.); clemens.becker@rbk.de (C.B.); 2 Institute of Epidemiology and Medical Biometry, Ulm University, Ulm 89081, Germany; 3 Department of Neuroscience, Norwegian University of Science and Technology, Trondheim NO-7491, Norway; alan.bourke@ntnu.no; 4 Institute of Movement and Sport Gerontology, German Sport University Cologne, Cologne 50933, Germany; zijlstra@dshs-koeln.de

**Keywords:** recovery, lie-to-standing transfer, inertial sensors, signal analysis, kinematic analysis, fall detection

## Abstract

Many older adults lack the capacity to stand up again after a fall. Therefore, to analyse falls it is relevant to understand recovery patterns, including successful and failed attempts to get up from the floor in general. This study analysed different kinematic features of standing up from the floor. We used inertial sensors to describe the kinematics of lie-to-stand transfer patterns of younger and healthy older adults. Fourteen younger (20–50 years of age, 50% men) and 10 healthy older community dwellers (≥60 years; 50% men) conducted four lie-to-stand transfers from different initial lying postures. The analysed temporal, kinematic, and elliptic fitting complexity measures of transfer performance were significantly different between younger and older subjects (i.e., transfer duration, angular velocity (RMS), maximum vertical acceleration, maximum vertical velocity, smoothness, fluency, ellipse width, angle between ellipses). These results show the feasibility and potential of analysing kinematic features to describe the lie-to-stand transfer performance, to help design interventions and detection approaches to prevent long lies after falls. It is possible to describe age-related differences in lie-to-stand transfer performance using inertial sensors. The kinematic analysis remains to be tested on patterns after real-world falls.

## 1. Background

Many older adults lack the capacity to stand up again after a fall [1]. Consequently, these people often remain on the floor, incapacitated for a long time, causing serious medical problems such as renal failure, pneumonia, dehydration or even death [2,3]. Clinicians and health-care providers have a strong interest in further investigating the circumstances of long-lies. One aspect is to develop autonomous technologies to detect these incidents [4] and to enable an accurate rescue chain [5]. Another aspect is to find indicators for a high risk of long lying periods after falling, to prevent these critical incidents by exercise interventions such as the backward chaining technique [6]. Thus, assessing standing up from a lying position could be a valuable approach. In a previous study [7] we described typical movement components and showed differences in terms of movement fragmentation during lie-to-stand transfers (LTS) of younger and older adults observed from video analyses. We expect that such differences will be reflected in a sensor based kinematic analysis of LTS which could help to assess the performance of lie-to-stand transfers. This could further help to identify people unable to stand up on their own and contribute to an understanding of successful recovery patterns after real-world falls. It was shown previously [4,8,9,10,11,12] that postural transitions from lying to sitting or standing postures can be detected from inertial sensors worn at the trunk. However, kinematic analysis to objectively describe and assess LTS performance from inertial sensor signals is yet to be investigated. Hence, this study aimed to assess selected kinematic features previously used for analysis of sit-to-stand transfers [13,14], such as vertical acceleration and rotational speed, and describe age-related differences during simulated LTS, based on inertial sensor signals collected with sensors worn at the trunk. A novel quantitative kinematic measure using a least square elliptic fitting method [15] was proposed to describe the complexity of individual movement patterns underlying LTS.

## 2. Methods

**Figure 1:**
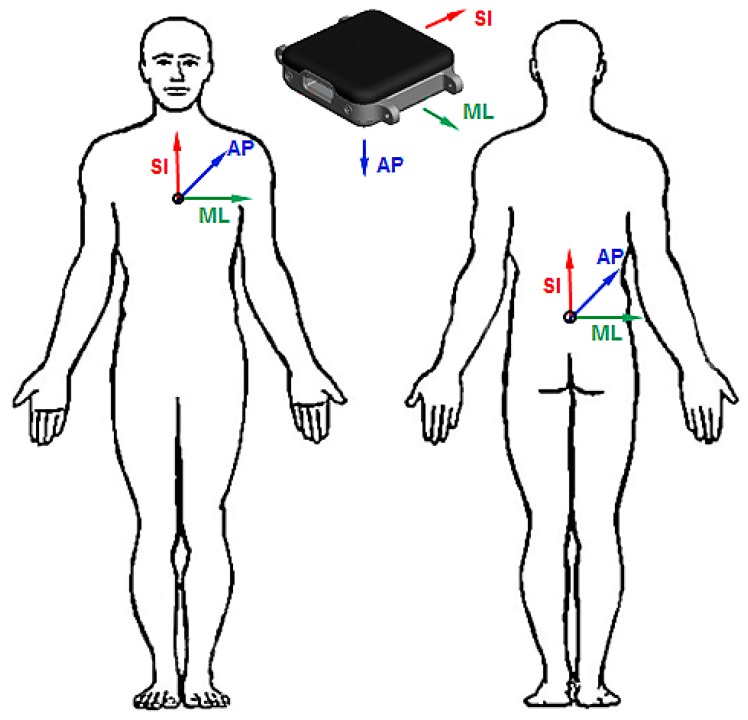
Wearable sensors fixed to the sternum and the L5 position. Medio-lateral axis (*ML*), anterior-posterior axis (*AP*), superior-inferior axis (*SI*).

Fourteen younger subjects (50% men) between 20 and 50 years of age and 10 healthy older community dwellers (≥60 years; 50% men) were included in this study. All participants were able to repeatedly stand up without help. Each participant was asked to conduct four LTS starting from different initial lying postures on the floor (lying on the back, front and both sides). The transfers were initiated voluntarily by the subjects who were asked to end the transfers in an erect standing position without considerable body movement. Kinematic data were recorded with Opal sensors (Figure 1, APDM, Portland, OR, USA) located on the trunk, including accelerometers, gyroscopes and magnetometers sampled at 128 Hz.

After processing the raw data output from the Opals, thresholds based on the average standard deviation were calculated from the three axes of the accelerometer and gyroscope signals recorded at the sternum and the L5 position from all subjects, to mark active periods (i.e., LTS). In a second step, the signals were filtered for periods including static postures where the subjects rested without performing movements. Periods of static posture were defined as listed in Table 1.

**Figure 2:**
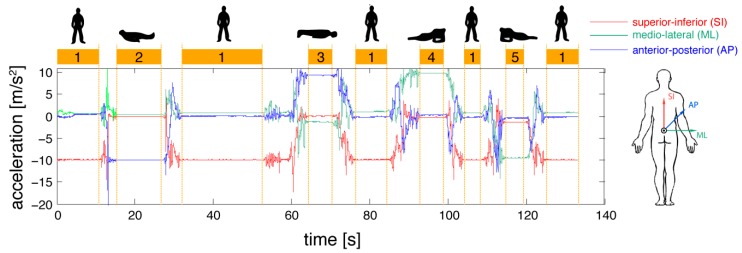
Separation between static postures and transfer movements from accelerometer signals.

Standing up from the floor was then classified with converse arguments, when a lying period (2, 3, 4 or 5) was followed by transfer movements and subsequent standing (1) as illustrated in Figure 2.

The following kinematic and temporal features were extracted to describe LTS and quantify age-related performance differences.

### 2.1. Transfer Duration

Based on the aforementioned classification system, start (*T_start_*) and end points (*T_end_*) of the transfers were calculated according to Equations (2) and (3) with *a_norm_* (*t*) being the time dependent norm acceleration as defined in Equation (1). A standard deviation threshold of the resultant acceleration (<0.15 m/s^2^) was obtained by visual inspection of all signals. For detection of the end point of the transfer, the same value was obtained. Additionally, the standard deviation of the angular velocity (0.1 rad/s) was included as a detection argument, in order to increase the robustness of detection. The transfer duration was then calculated according to Equation (4):

(1) $$a_{norm}\left(t\right)=\sqrt{a_{SI}{\left(t\right)}^{2}+a_{AP}{\left(t\right)}^{2}+a_{ML}{\left(t\right)}^{2}}$$

(2) $$T_{start}=\begin{array}{c} \{t,a_{norm}\left(t\right)\}<0.15{m/s}^{2} \end{array}$$

(3) $$T_{end}=\begin{array}{c} \{t,a_{norm}\left(t\right)\}<0.15{m/s}^{2}\wedge \omega \left(t\right)<0.1\;\mathrm{rad}/s \end{array}$$

(4) $$T_{rising}=T_{end}-T_{start}$$

### 2.2. Transfer Angular Velocity (Root Mean Square of Rotational Speed, RMS)

For each axis of rotation, the *RMS* of angular velocities were calculated according to Equations (5)–(7), with the time dependent gyroscope signals of the single axes referred to as ω and the sample frequency of the recordings as *ƒ_S_*. The total *RMS* was calculated based on the arithmetic mean of *RMS_SI_*, *RMS_AP_*, and *RMS_ML_*. This value characterises the movement velocity during the transfer.

(5) $$RMS_{SI}=\sqrt{\frac{\sum_{t=T_{start}}^{T_{end}}\omega_{SI}{\left(t\right)}^{2}}{f_{s}\cdot T_{rising}}}$$

(6) $$RMS_{AP}=\sqrt{\frac{\sum_{t=T_{start}}^{T_{end}}\omega_{AP}{\left(t\right)}^{2}}{f_{s}\cdot T_{rising}}}$$

(7) $$RMS_{ML}=\sqrt{\frac{\sum_{t=T_{start}}^{T_{end}}\omega_{ML}{\left(t\right)}^{2}}{f_{s}\cdot T_{rising}}}$$

### 2.3. Vertical Acceleration

Based on the assumption that the horizontal acceleration component within transfers will be rather small relative to the vertical component due to the contribution of gravity, which was shown for sit-to-stand transfers in previous work [14]. Vertical acceleration for each time point (*t*) within the LTS was estimated from the norm acceleration Equation (1) since it is strongly defined by the vertical movement component. The residual of the norm acceleration was then calculated from the filtered norm acceleration signal (2nd order Butterworth low-pass filter with cut-off frequency of 3 Hz) with the gravity component (9.81 m/s^2^) being subtracted (Equation (8)). The maximum value extracted from the time series of LTS of norm acceleration was then analysed.

(8) $$a_{vert\;norm}\left(t\right)=a_{norm\_filtered}\left(t\right)-g$$

### 2.4. Vertical Velocity

The vertical velocity of the trunk within the LTS was calculated by integrating the norm acceleration according to Equation (9):

(9) $$v_{vert}=\overset{T_{end}}{\underset{t=T_{start}}{\sum }}a_{vert\;norm}\left(t\right)$$

### 2.5. Jerk

Jerk was defined as the change in the acceleration signals calculated for each axis Equations (10)–(12), for the interval between the start and the end of the transfers. Total Jerk (J) was calculated based on the arithmetic mean of *J_SI_*, *J_AP_*, and *J_ML_*; the maximum value of the Total Jerk time series (*J_Max_*) was analysed:

(10) $$J_{SI}\left(k\right)=f_{s}\frac{a_{SI,k+1}-a_{SI,k-1}}{2},with\;k=f_{s}T_{start},\ldots ,f_{s}T_{end}$$

(11) $$J_{AP}\left(k\right)=f_{s}\frac{aAP_{,k+1}-a_{AP,k-1}}{2},with\;k=f_{s}T_{start},\ldots ,f_{s}T_{end}$$

(12) $$J_{ML}\left(k\right)=f_{s}\frac{a_{ML,k+1}-a_{ML,k-1}}{2},with\;k=f_{s}T_{start},\ldots ,f_{s}T_{end}$$

## 3. Smoothness

Smoothness, calculated to describe whether the movement sequence was continuous and linear within a clear direction, was presented as a measure for skilled coordinated movement in the work of Bagalà and colleagues [13]. Therefore, according to the suggestion of Hogan and Sternad [16], the jerk of the single axes was summed up and normalised by the transfer duration according to Equations (13)–(15). Total Smoothness (*S*) was calculated based on the arithmetic mean of *S_SI_*, *S_AP_*, and *S_ML_*. It was hypothesised that the smoothness in the younger age group will present higher values and the range of variation will be greater in the older subject group due to greater heterogeneity.

(13) $$S_{SI}={T_{rising}}^{3}\overset{T_{end}}{\underset{t=T_{start}}{\sum }}\left|J_{SI}\left(t\right)\right|$$

(14) $$S_{AP}={T_{rising}}^{3}\overset{T_{end}}{\underset{t=T_{start}}{\sum }}\left|J_{AP}\left(t\right)\right|$$

(15) $$S_{ML}={T_{rising}}^{3}\overset{T_{end}}{\underset{t=T_{start}}{\sum }}\left|J_{ML}\left(t\right)\right|$$

### 3.1. Fluency

As a measure of the steadiness of bodily motions separated from gravity induced acceleration components [13], the fluency was calculated based on the difference between the raw signals and the filtered signals (second order Butterworth low-pass filter, cut-off frequency 3 Hz) for each axis of acceleration according to Equations (16)–(18):

(16) $$Fl_{SI}={T_{rising}}^{2}\overset{T_{end}}{\underset{t=T_{start}}{\sum }}\left|a_{SI}\left(t\right)-a_{SI_{\_filtered}}\left(t\right)\right|$$

(17) $$Fl_{AP}={T_{rising}}^{2}\overset{T_{end}}{\underset{t=T_{start}}{\sum }}\left|a_{AP}\left(t\right)-a_{AP\_filtered}\left(t\right)\right|$$

(18) $$Fl_{ML}={T_{rising}}^{2}\overset{T_{end}}{\underset{t=T_{start}}{\sum }}\left|a_{ML}\left(t\right)-a_{ML\_filtered}\left(t\right)\right|$$

The absolute difference was then summed up for, and normalised with, the transfer duration. Total fluency (Fl) was calculated based on the arithmetic mean of the three axes Fl_SI_, Fl_AP_, and Fl_ML_.

### 3.2. Complexity of Movement Strategies (Elliptic Fitting)

In a first step, quaternion values were extracted from the sensor data delivered by the commercial APDM sensor system using validated algorithms. Quaternions were then transformed into Euler angles to define global sensor orientation. When the subject rested on the ground during the lying components before initiating the transfers, Euler angles were set to zero. Subsequently, the change of orientation within the different axes (*SI, AP, ML*) of the sensor located at the lower back (L5) was calculated. Based on angular rotation plots of continuous movements of the complete LTS, a least square elliptic fitting method [15] was used to describe the complexity of different transfer strategies from lying to standing for each patient. This algorithm fits a 2D point cloud of angular rotation around the *ML*, *SI* and *AP* axes into ellipses. It was hypothesised that the continuous trajectory of angular rotation change within the LTS resembles an ellipse. Therefore, angular rotation was plotted in pairwise combinations of all axes (*ML-AP, ML-SI, SI-AP*). Smaller width (length of transversal axis) and height (length of longitudinal axis) of the ellipses sought to explain greater motion steadiness in terms of rotational movement around the axes. The angle between the longitudinal axes of the *ML-SI* and the *ML-AP* ellipses sought to explain the linearity of the movement (90° in linear movement patterns) and reveal differences in complexity of transfer strategies. A stronger deviation from a linear 90° pattern in older subjects was hypothesised. This hypothesis was based on different movement patterns within the motion sequences of younger and older subjects standing up from the floor, including variable use of rotational trunk movement observed from video-footage in a previous study of our group [7].

All calculations were performed using MATLAB R2014a (The MathWorks, Inc., Natick, MA, USA). Statistics were processed using SPSS 16 (SPSS Inc., Chicago, IL, USA). Due to the small sample size, non-parametric statistics were applied. Wilcoxon tests were used to describe differences between the younger and the older subject group. The study was approved by the Ethical Committee at the University Hospital Tuebingen (No. 212/2013BO2). All participants gave written informed consent prior to testing.

## 4. Results

Ninety-six LTS from different initial lying postures were classified from accelerometer and gyroscope data from the trunk sensors for kinematic analysis. The analysed kinematic and temporal parameters describing and assessing standing up from different lying postures (mean of standing up from lying on the back, front and both sides), including transfer duration, transfer velocity, maximum vertical acceleration and velocity as well as fluency and smoothness of movement, discriminated between the younger and older subject group, independent of the sensor location at the trunk (L5 or Sternum, Table 2). The calculations based on sternum sensor signals showed slightly different median values compared to values detected from L5 sensor signals for all parameters.

A significant difference in rotational transfer velocity (total root mean square of rotational speed, RMS) between the age groups was found for the mean values of all positions (*p* = 0.001 Sternum, *p* = 0.007 L5). The highest difference regarding the single lying postures before transfer initiation was observed for standing up from lying on the back (92.0°/s, older 291.0°/s and younger 199°/s) measured at the sternum position. The lowest maximal vertical acceleration that was sufficient to successfully stand up from the floor in older subjects in this study was at 1.8 m/s^2^ (from lying on the back, Sternum) and 1.2 m/s^2^ (from lying on the back, L5). The lowest maximal vertical velocity was at 0.3 m/s (from lying on the left side, Sternum) and 0.3 m/s^2^ (from lying on the right side, L5).

**Figure 3:**
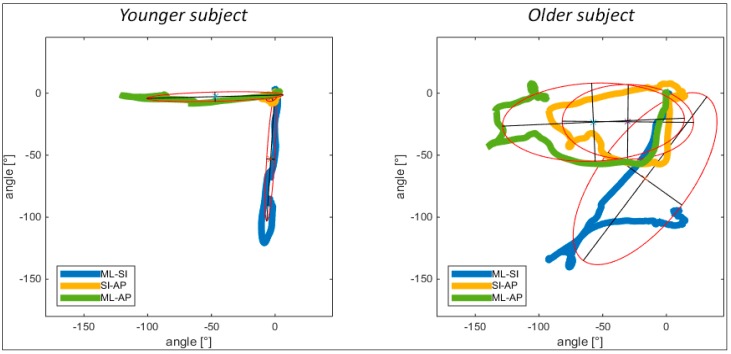
Different complexity levels and transfer strategies of a younger and older subject displayed using the ellipse fitting method, with ellipses indicating 2D plots of rotational movement around combined axes (*SI, AP, ML*). Axes of ellipses: longitudinal blue = *ML*, transversal blue = I; longitudinal yellow = *SI*, transversal yellow = *AP*; longitudinal green = *ML*, transversal green = *AP*.

Orientation data was available for *n* = 15 subjects (nine younger subjects, 6 older subjects). Motion steadiness was higher in the younger subjects, as expressed in narrow ellipses compared to the older subjects. Rotational movement in *SI-AP* axes hardly existed, hence, very small ellipses were presented (Figure 3). In the older subjects more complex patterns, expressed by extensively broad and high ellipses were observed, including also the *SI-AP* ellipse. The angle between the longitudinal axes of the *ML-SI* and *ML-AP* ellipses was close to 90° in the younger subjects while the angle in the older subjects was considerably smaller (roughly 45°).

Quantitative results of the ellipse fitting analysis are listed in Table 3. Both ellipse width and height showed significantly higher values for the *ML-SI* as well as the *SI-AP* ellipses within the older subject group, indicating the requirement to use more rotational movement strategies when standing up from lying on the back. Furthermore, the deviation of the angle between the longitudinal axes of the *ML-SI* and *ML-AP* ellipses from the optimal 90° angle was significantly higher in the older subject group, indicating less linear movement patterns. This analysis showed the usefulness of the ellipse measures, to discriminate between different levels of complexity in rising performances.

## 5. Discussion

The selected kinematic and temporal parameters in this study described and discriminated the LTS performance from different lying postures of younger and older subjects. Our findings confirmed the feasibility to analyse different movement patterns underlying LTS for signals recorded with sensors worn at the trunk. However, movement components in which the arms and legs were positioned for supporting the body weight mostly contained small amounts of trunk movement, thus these movements could not be recognised reliably.

As shown in Table 2, significant differences between younger and older subjects were observed in almost all performance parameters of LTS. These results give insight into biomechanical features when standing up from lying on the floor. This may, within a certain scope, help to understand mechanisms of successful recovery strategies after falls. Besides the development of more extensive fall detection approaches, this could be helpful for physiotherapists to design tailored training interventions. Interventions could for example include training of more rotational movement patterns that were shown to be helpful for older people in standing up from the floor. Furthermore, quantification and assessment of LTS performance addresses clinical interest for being a potential indicator for the inability to stand up thus having a higher risk of long lies after falls. A simple and appropriate measure to quantify the LTS performance of younger and older subjects was the transfer duration. The results showed longer transfer durations at the sternum position compared to the L5 position. This can be explained by using the same thresholds to define start and end points of transfers at both positions, with the sternum positions being exposed to higher momentum and earlier movement initiation due to the longer distance from the centre of mass. Though it was feasible to measure the transfer duration at the L5 position, it is recommended to use a sensor worn at the sternum position for a more exact estimation. Another reasonable modification of the estimation of the transfer duration might be the use of individualised thresholds. Fixed thresholds were seen to negatively influence the precision in estimating end-points of the transfers, due to more intense body sway, received by the trunk sensors during stabilisation after righting up in older subjects. However, due to the limited dataset, the results remain to be confirmed by further analysis including more subject data.

Beyond the temporal parameters, significant differences were observed for maximal vertical acceleration and vertical velocity, likely explained by different movement patterns. Previous observations from video-analyses [7] showed that older persons tend to reposition their hands and feet more often than younger subjects during elevation movements, thus splitting up elevation into several components. These broken movement patterns could be an explanation for lower vertical acceleration and velocity values found in this study, which requires further analysis. Repositioning of the hands and feet as seen from the video footage could not be reliably analysed from sensor signals in this study. However, sensor analyses helped overcome subjective estimation of start and end positions and gave insight into performance quality parameters. Maximum vertical acceleration measured at the sternum position may be a valuable parameter to assess LTS performance. The norm acceleration based method to calculate vertical acceleration and vertical velocity was deemed sufficient since the horizontal acceleration did not affect the trend of the results, which was in line with the findings of Zhang and colleagues on sit-to-stand transfers [14]. The lowest maximal vertical acceleration and velocity values during LTS in older subjects presented in this study could serve as a reference for further analysis to develop threshold based detection approaches of recovery after falls. However, the presented values remain to be compared to those from real-world recovery patterns in older subjects with impaired LTS capacity.

The motion sequences observed in the older subject group were significantly less fluent and smooth than in the younger group, indicating more difficulties in performing steady movements. Comparable results were outlined by Bagalà and colleagues [13] for older patients standing up from lying on a hospital bed. Again, the much higher values calculated for the sternum sensor in comparison to the L5 sensor probably resulted from higher sensitivity to upper trunk movement, as well as the dependency on different transfer durations measured at the L5 compared to the sternum position. It was further shown that older subjects stood up with significantly higher rotational transfer velocity (RMS), especially when standing up from lying on the back, which was an indicator for an increase in the use of rotational movement patterns in all three axes, possibly indicating compensational movement patterns to prevent peak loads of the muscular system. The change of acceleration within the movements, as indicated by the maximum jerk, only showed the tendency to differ but failed to reach significance. This might on the one hand be the result of the low number of subjects but may also be due to the uncertainty in whether maximum jerk was generated within the pre-elevation phase, which could contain higher rotational movement in older subjects, or within the elevation phase. Bagalà and colleagues [13] instead showed good correlation between jerk, smoothness and fluency. The difference could have resulted from the different conditions when standing up from a higher levelled hospital bed, where it is unnecessary to intensively prepare for elevation involving jerky movement behaviour. These findings suggest further kinematic analyses of separate movement components, such as pre-elevation and elevation component as described in [7], are valuable.

As shown in Table 3, significant differences were observed between younger and older subjects in terms of complexity of the movement patterns. Higher values were observed for the length and width of the ellipses, indicating greater difficulty as described by the need to use more indirect movement strategies including more turns around the longitudinal axis to prepare for elevation in the older subjects. The shapes of the ellipses showed that younger subjects tended to use less rotational trunk movement in the *SI* and *AP* axes and predominantly rotated around the *ML* axis. In contrast, the older subjects tended to primarily rotate around the *SI* axis to roll into a side-lying position, which was applied with a more complex movement pattern, as visualised with the trajectories fitted into the ellipses (Figure 3). Similarly, the significantly different angles between the *ML-SI* and *ML-AP* ellipses showed a decreased linearity of the motion sequence in the older subject group. The presented ellipse-fitting measure was deemed a valid descriptor of complexity of the LTS performance. However, it relied on the orientation estimation derived from the commercial APDM sensor system. Further analysis has to be conducted to confirm the orientation estimation using pre-defined methodology, such as an extended Kalman filter [17] or sensor fusion approach [18]. This will further help to define the acceleration and velocity of the trunk segment during different phases of the LTS in the vertical direction, to improve the precision of norm acceleration based results.

A methodological limitation existed due to accelerometer drift, violating the calculation of maximal vertical velocity. The drift was reduced by integrating the signals starting from the end-points of the transfers. This method was chosen after observing that elevation phases, including maximal vertical velocity, were mostly located at the end of the motion sequences. Furthermore, a linear drift compensation method was used to calculate the transfer velocity, which seemed reasonable due to rather short transfer intervals in this study. A further limitation was that the data set only included voluntary healthy subjects and did not contain unsuccessful LTS attempts of older people or real-world fall data. Therefore, it remains to be investigated whether the performance parameters, shown in this study to describe and discriminate the performance of younger and older adults, are eligible to identify a high risk for long lying. LTS were assessed with the subjects initially resting on the floor without moving the trunk, which might not be representative for real-world recovery patterns after a fall. Even within long lying periods, the faller might attempt to stand up or at least orient to a sitting position several times without recovering successfully. Further kinematic analysis will be needed to describe successful and unsuccessful recovery patterns after real-world falls. This will be the focus of the FARSEEING group [19] who set up a real-world fall repository including data from inertial sensors.

## 6. Conclusions

In conclusion, this study showed the feasibility of describing and discriminating the performance kinematics of younger and older subjects standing up from the floor from different lying postures, calculated from inertial sensor signals recorded at the trunk. The extracted kinematic features gave insight into different movement patterns of LTS and could help to assess different recovery scenarios after real-world falls, design interventions and develop detection approaches to prevent long lies after falls.

## Figures and Tables

**Table 1:** Definitions for static posture periods.

Code	Static Posture	Classification Arguments
(1)	Standing	(acc_SI_ < −5 m/s^2^) AND (acc_SI_ < acc_ML_) AND (acc_SI_ < acc_AP_) AND (static posture = true)
(2)	Lying on the back	(acc_AP_ > 5 m/s^2^) AND (acc_AP_ > acc_SI)_ AND (acc_AP_ > acc_ML_) AND (static posture = true)
(3)	Lying on the front	(acc_AP_ < 5 m/s^2^) AND (acc_AP_ < acc_SI)_ AND (acc_AP_ < acc_ML_) AND (static posture = true)
(4)	Lying on the left side	(acc_ML_ < −5 m/s^2^) AND (acc_ML_ < acc_SI)_ AND (acc_ML_ < acc_AP_) AND (static posture = true)
(5)	Lying on the right side	(acc_ML_ > −5 m/s^2^) AND (acc_ML_ > acc_SI)_ AND (acc_ML_ > acc_AP_) AND (static posture = true)

**Table 2:** Basic quantitative parameters to describe standing up from different lying postures (back, front, side left and right, mean of four transfers) from trunk sensors (L5, Sternum) in younger and older subjects.

*Feature Parameters*	Sensor	*Young Subjects (n = 14)*		*Older Subjects (n = 10)*		*p*
		Median (Q1–Q3 ^a^)	Min–Max	Median (Q1–Q3 ^a^)	Min–Max	
Transfer Duration (s)	L5	4.2 (3.8–5.2)	3.1–6.0	6.0 (5.5–7.0)	4.7–8.9	0.000
	ST	4.5 (4.0–5.4)	3.3–6.4	6.3 (5.7–7.9)	5.1–9.9	0.000
Transfer Velocity ^b^ (°/s)	L5	163.6 (139.5–178.4)	127.0–251.4	206.7 (169.2–236.3)	164.2–285.2	0.007
	ST	210.3 (196.2–233.8)	163.1–293.3	272.6 (250.8–296.0)	219.7–395.6	0.001
Max. Vertical Acceleration (m/s^2^)	L5	4.7 (4.1–6.1)	2.8–6.8	3.5 (2.6–4.5)	1.7–4.6	0.009
	ST	5.9 (4.7–6.5)	4.1–8.7	3.6 (3.3–4.4)	2.0–4.7	0.000
Max. Vertical Velocity (m/s)	L5	1.1 (0.9–1.2)	0.7–1.4	0.8 (0.6–1.0)	0.2–1.1	0.011
	ST	1.2 (1.1–1.5)	0.9–2.0	1.0 (0.8–1.4)	0.6–1.4	0.036
Maximum Jerk (J_Max_) (m/s^3^)	L5	67.0 (46.2–89.2)	36.4–383.3	41.0 (30.6–62.6)	18.3–99.2	0.056
	ST	71.0 (44.2–107.6)	19.8–356.7	40.9 (34.8–61.2)	30.4–121.4	0.056
Smoothness (S) × 10^6^ (m)	L5	5.9 (5.7–6.0)	5.3–6.5	6.4 (6.2–6.6)	6.1–6.9	0.000
	ST	1.0 (0.7–1.7)	0.5–3.8	3.2 (2.4–7.8)	1.6–18.8	0.000
Fluency (Fl) × 10^3^ (m)	L5	4.0 (3.8–4.1)	3.6–4.4	4.4 (4.2–4.5)	4.1–4.7	0.000
	ST	12.4 (8.9–18.3)	7.0–32.2	30.9 (22.6–49.8)	17.8–100.5	0.000

^a^ Interquartile range (quartile 25%–75%); ^b^ Total root mean square of rotational speed.

**Table 3:** Quantitative results of the ellipse fitting analysis of younger and older subjects standing up from lying on the back.

*Ellipse Fitting Complexity Measures*	Axes	*Younger Subjects (n = 9)*		*Older SUBJECTS (n = 6)*		*p*
		Median (Q1–Q3)	Min–Max	Median (Q1–Q3)	Min–Max	
Ellipse Width (°)	ML-AP	22.5 (10.1–28.0)	6.1–35.6	35.8 (26.1–58.3)	24.1–62.9	0.012
	AP-SI	20.7 (8.1–25.9)	5.4–30.9	36.3 (31.0–61.6)	30.5–65.9	0.001
	ML-SI	24.3 (14.7–42.8)	6.3–66.8	65.3 (58.4–73.4)	55.4–78.1	0.005
Ellipse Height (°)	ML-AP	120.9 (103.1–136.6)	95.1–166.4	146.6 (127.5–170.1)	103.4–209.4	0.113
	AP-SI	30.0 (16.7–58.7)	8.8–114.2	94.9 (84.0–144.8)	81.7–226.2	0.008
	ML-SI	117.0 (100.1–128.0)	87.0–165.0	151.0 (134.2–205.0)	129.0–278.0	0.008
Deviation of angle between ellipses from 90° (°)	ML-SI to ML-AP	7.0 (3.5–19.5)	2.0–38.0	41.5 (30.5–46.3)	29.0–50.0	0.002

*p*-Values based on Wilcoxon test.

## References

[1] Gurley R.J., Lum N., Sande M., Lo B., Katz M.H. (1996). Persons found in their homes helpless or dead. N. Engl. J. Med..

[2] Fleming J., Brayne C., Cambridge City over-75 s Cohort (CC75C) Study Collaboration (2008). Inability to get up after falling, subsequent time on floor, and summoning help: Prospective cohort study in people over 90. BMJ.

[3] Lord S.R., Sherrington C., Menz H.B. (2003). Falls in Older People: Risk Factors and Strategies for Prevention. Inj. Prev..

[4] Karantonis D.M., Narayanan M.R., Mathie M., Lovell N.H., Celler B.G. (2006). Implementation of a real-time human movement classifier using a triaxial accelerometer for ambulatory monitoring. IEEE Trans. Inf. Technol. Biomed..

[5] Redmond S.J., Zhang Z., Narayanan M.R., Lovell N.H. Pilot evaluation of an unobtrusive system to detect falls at nighttime. Proceedings of the 36th Annual International Conference of the IEEE Engineering in Medicine and Biology Society.

[6] Hofmeyer M.R., Alexander N.B., Nyquist L.V., Medell J.L., Koreishi A. (2002). Floor-rise strategy training in older adults. J. Am. Geriatr. Soc..

[7] Schwickert L., Oberle C., Becker C., Lindemann U., Klenk J., Schwenk M., Bourke A.K., Zijlstra W. (2015). Model development to study strategies of younger and older adults getting up from the floor. Aging Clin. Exp. Res..

[8] Najafi B., Aminian K., Paraschiv-Ionescu A., Loew F., Büla C.J., Robert P. (2003). Ambulatory system for human motion analysis using a kinematic sensor: Monitoring of daily physical activity in the elderly. IEEE Trans. Biomed. Eng..

[9] Paraschiv-Ionescu A., Buchser E.E., Rutschmann B., Najafi B., Aminian K. (2004). Ambulatory system for the quantitative and qualitative analysis of gait and posture in chronic pain patients treated with spinal cord stimulation. Gait Posture.

[10] Allen F.R., Ambikairajah E., Lovell N.H., Celler B.G. An adapted Gaussian mixture model approach to accelerometry-based movement classification using time-domain features. Proceedings of the 28th Annual International IEEE Conference on Engineering in Medicine and Biology Society.

[11] Khan A.M., Lee Y.-K., Lee S.Y., Kim T.-S. (2010). A triaxial accelerometer-based physical-activity recognition via augmented-signal features and a hierarchical recognizer. IEEE Trans Inf. Technol. Biomed..

[12] Mortazavi B., Nemati E., VanderWall K., Flores-Rodriguez H.G., Cai J.Y.J., Lucier J., Naeim A., Sarrafzadeh M. (2015). Can Smartwatches Replace Smartphones for Posture Tracking?. Sensors.

[13] Bagalà F., Klenk J., Cappello A., Chiari L., Becker C., Lindemann U. (2013). Quantitative description of the lie-to-sit-to-stand-to-walk transfer by a single body-fixed sensor. IEEE Trans. Neural Syst. Rehabil. Eng..

[14] Zhang W., Regterschot G., Schaabova H., Baldus H., Zijlstra W. (2014). Test-Retest Reliability of a Pendant-Worn Sensor Device in Measuring Chair Rise Performance in Older Persons. Sensors.

[15] Fitzgibbon A., Pilu M., Fisher R.B. (1999). Direct least square fitting of ellipses. IEEE Trans. Pattern Anal. Mach. Intell..

[16] Hogan N., Sternad D. (2009). Sensitivity of Smoothness Measures to Movement Duration, Amplitude, and Arrests. J. Mot. Behav..

[17] Sabatini A.M. (2011). Kalman-filter-based orientation determination using inertial/magnetic sensors: Observability analysis and performance evaluation. Sensors.

[18] Valenti R.G., Dryanovski I., Xiao J. (2015). Keeping a Good Attitude: A Quaternion-Based Orientation Filter for IMUs and MARGs. Sensors.

[19] FARSEEING. www.farseeingresearch.eu.

